# Case of Penetrating Gun-shot Wound of the Heart

**Published:** 1855-09

**Authors:** 


*Case of Penetrating Gun Shot Wound of the Heart.*—The above case is reported by Dr. Carnochan of N. York in pamphlet form, although it appeared previously in the *American Medical Monthly*. It is accompanied by an accurate drawing, constituting it a very valuable illustration.

There is nothing very remarkable that a ball should lodge in the muscular structure of the heart; this might have and doubtless has, occurred again and again, but the case is interesting because life was prolonged for eleven days, during a part of which time the patient betrayed but little manifestation of suffering or even irritation. The autopsy showed that the opening made by the ball into the pericardium was closed by plastic exudation, that the cavity was filled with serum slightly tinged with blood and so much distended as to encroach upon the lungs on either side. The heart in *situ* gave at first sight no evidence of the existence, within its substance, of a foreign body of any kind, but the sense of touch detected a ball in the muscular tissue, one third of an inch in diameter, embedded in a cyst one-fourth of an inch beneath the outer surface and in the septum between the right and left ventricles.

The cause of death is attributable to the inflammation and its resulting consequences.

The case is a peculiar one because of the little disturbance it occasioned for some days and the duration of life after the accident. Evidently nature was making an effort at reconciliation and cure for the opening into the pericardium was provided for and the ball was well encysted. Its position, too, was fortunate for the free action of the heart, and could the inflammation of the pericardium have been controlled, we see no reason why the individual might not have lived for years. The heart was not embarrassed by the pressure of the foeign body, for the pulse was regular and slow for nine days, during which it did not exceed 84 at any time but generally slower. On the 10th day the symptoms changed from those decidedly encouraging to those of graver import which increased in gravity until he died.

The patient in this case was the notorious William Poole, and the circumstances under which the injuries were received, added much to swell the notoriety of the case and awaken a curiosity and an interest as to the nature of the injury.

*Tanner's Manual of Clinical Medicine.* Blanchard & Lea, Philadelphia.

We have received this choice little work, which is well calculated to answer a requirement on the part of the profession. It is intended for daily reference, and to aid in the study of disease at the bed-side. The first section is upon the "faculty of observa tion," the second, "the general conduct of the practitioner of medicine," the third, the "clinical examination of the patient," &c., and finally closes with the "Code of Ethics adopted by the American Medical Association."

This valuable aid to the student and practitioner is so small as to be carried readily in the pocket, and while perusing it the thought occurred to us how profitably the time of the practitioner could be employed in its perusal when detained away from his home in a case which did not require his immediate attention. That the hours may not drag heavily, he is oftimes forced to employ them upon some of the light valueless productions now so largely published and generally read. In "Tanner's Manuel," however, he will find much of substantial value and worthy of remembrance.

*Caution.*—Persons desirous of attending the Lectures of the "College of Physicians and Surgeons of the Iowa University" will if they desire to avail themselves of the benefits of Scholarships, apply to the Members of the Legislature of this State, and to other

